# One Health: Circadian Medicine Benefits Both Non-human Animals and Humans Alike

**DOI:** 10.1177/07487304241228021

**Published:** 2024-02-20

**Authors:** Hesham I. Farag, Barbara A. Murphy, James R. Templeman, Charlene Hanlon, Jessica Joshua, Thomas G. Koch, Lee Niel, Anna K. Shoveller, Gregoy Y. Bedecarrats, Amy Ellison, David Wilcockson, Tami A. Martino

**Affiliations:** * Department of Biomedical Sciences, Ontario Veterinary College, University of Guelph, Guelph, ON, Canada; † Centre for Cardiovascular Investigations, University of Guelph, Guelph, ON, Canada; ‡ School of Agriculture and Food Science, University College, Dublin, Ireland; § Department of Animal Biosciences, University of Guelph, Guelph, ON, Canada; || Department of Poultry Science, Auburn University, Auburn, Alabama, USA; ¶ Department of Clinical Studies, Ontario Veterinary College, University of Guelph, Guelph, ON, Canada; # Department of Pathobiology, University of Guelph, Guelph, ON, Canada; ** School of Natural Sciences, Bangor University, Bangor, UK; †† Department of Life Sciences, Aberystwyth University, Aberystwyth, UK

**Keywords:** circadian medicine, circadian, veterinary, animal health, human health, companion animals, dogs, cats, horses, chickens, time-restricted feeding, hospitals, intensive care unit, animal welfare, aquaculture

## Abstract

Circadian biology’s impact on human physical health and its role in disease development and progression is widely recognized. The forefront of circadian rhythm research now focuses on translational applications to clinical medicine, aiming to enhance disease diagnosis, prognosis, and treatment responses. However, the field of circadian medicine has predominantly concentrated on human healthcare, neglecting its potential for transformative applications in veterinary medicine, thereby overlooking opportunities to improve non-human animal health and welfare. This review consists of three main sections. The first section focuses on the translational potential of circadian medicine into current industry practices of agricultural animals, with a particular emphasis on horses, broiler chickens, and laying hens. The second section delves into the potential applications of circadian medicine in small animal veterinary care, primarily focusing on our companion animals, namely dogs and cats. The final section explores emerging frontiers in circadian medicine, encompassing aquaculture, veterinary hospital care, and non-human animal welfare and concludes with the integration of One Health principles. In summary, circadian medicine represents a highly promising field of medicine that holds the potential to significantly enhance the clinical care and overall health of all animals, extending its impact beyond human healthcare.

## Introduction

The health of soil, plant, animal and man is one and indivisible (Albert Howard).

Early breakthroughs in circadian biology emerged from studies involving non-human animals. Researchers like Patricia DeCoursey, investigating diurnal behaviors in wild-caught rodents like squirrels and chipmunks, coupled with laboratory-bred animal studies, notably contributed to our understanding of mammalian circadian biology (DeCoursey, 1960, 1964, 1986b, 1986a; DeCoursey et al., 1997; DeCoursey and Krulas, 1998; DeCoursey et al., 1998, 2000; DeCoursey, 2014). Nicholas Mrosovsky (1971) explored the circadian biology of ground squirrels, and later investigated daily rhythmicity in laboratory hamsters (Shibuya et al., 1980). Studies on wild birds such as house sparrows and migrant songbirds furthered our understanding of avian circadian rhythms (Gwinner et al., 1997; Cassone, 2014). Foundational research such as these, and many others such as those described below, advanced our knowledge of circadian rhythms in non-human animals and paved the way for broader implications in human medical advancements, ultimately paving the way for the emergence of circadian medicine.

A pivotal component of circadian biology revolves around melanopsin, a photopigment localized in distinct retinal ganglion cells separate from rods and cones. The identification of the melanopsin gene by Provencio and colleagues (1998), its presence in the human retina (Provencio et al., 2000), and its association with intrinsically photosensitive retinal ganglion cells (ipRGCs; Lucas et al., 1999; Berson et al., 2002; Hattar et al., 2002) significantly influenced our understanding. Melanopsin’s sensitivity to short-wavelength blue light in humans (Brainard et al., 2001; Thapan et al., 2001) underscores its role in synchronizing our internal body clock with the light-dark (L:D) cycle and regulating core circadian processes like melatonin production (Arendt, 2019). Extensive research into light’s influence on circadian biology has been pivotal for understanding its broader orchestration of physiological functions (Bedrosian et al., 2013; Fonken and Nelson, 2014; Nelson et al., 2021).

Molecular circadian mechanisms govern 24 h daily cellular cycles and are present in virtually all our cells. These mechanisms, including factors like brain and muscle ARNT-like 1 (BMAL1), circadian locomotor output cycles kaput, cryptochrome, period (PER1/2/3), nuclear receptor Subfamily 1 Group D Member 1 (NR1D2; REV-ERBa/b), and retinoic acid receptor–related orphan receptor, oscillate daily in the SCN, extending to peripheral clocks in different tissues, contributing to overall physiological coordination. Microarray studies have extensively documented these rhythmic cycles (e.g., Panda et al., 2002; Storch et al., 2002; Martino et al., 2004; Rudic et al., 2005) as have studies using other techniques, with similar findings in non-human primates (Mure et al., 2018) and human organs like the heart (Leibetseder et al., 2009) and others (Zhang et al., 2014; Hughes et al., 2017; Ruben et al., 2018). The molecular circadian mechanism has been extensively reviewed, for example (Roenneberg and Merrow, 2005; Eckel-Mahan et al., 2012; Martino and Young, 2015; Takahashi et al., 2018; Rabinovich-Nikitin et al., 2021).

While variations exist across species, with species-specific differences highlighted in each of the relevant sections below, it is generally noted that circadian rhythms are involved in many vital body functions, impacting heart rate, blood pressure, body temperature, hormone production, metabolism, activity, and autonomic nervous system bias. Research and reviews in circadian biology have extensively covered these aspects (Reppert and Weaver, 2002; Roenneberg and Merrow, 2005; Martino and Sole, 2009; Sole and Martino, 2009; Alibhai et al., 2015; Martino and Young, 2015; Mistry et al., 2017; Khaper et al., 2018; Rabinovich-Nikitin et al., 2019; Gumz et al., 2023), including sleep’s partial regulation by circadian rhythms alongside homeostatic control (Meyer et al., 2022; Hastings et al., 2023). Understanding circadian rhythms across animal groups provides insights into their evolutionary adaptations, forming a foundation for applying circadian principles to enhance the health and welfare of all animals.

Despite being primarily focused on human health outcomes (e.g., Martino and Sole, 2009; Alibhai et al., 2015; Reitz and Martino, 2015; Cederroth et al., 2019; Martino and Harrington, 2020; Allada and Bass, 2021; Aziz et al., 2021; Kramer et al., 2022; Gumz et al., 2023; Martino and Delisle, 2023; Sole and Martino, 2023), circadian medicine has largely overlooked its potential impact on non-human animals. This review aims to redirect attention to circadian medicine’s principles that could significantly benefit companion animals, agricultural livestock, and animal husbandry practices. The term “animal” in this context generally refers to non-human animals unless otherwise specified. By examining foundational studies and exploring their implications, we can unlock the potential for developing medical applications based on circadian rhythms across diverse species.

## Health and welfare benefits of circadian medicine for animals

Investigating circadian medicine for non-human domesticated animals holds immense promise for enhancing their health and welfare. This potential is rooted in the sheer number of domesticated animals integrated into our societies. Annually, over 70 billion animals are managed in farm settings worldwide, including 59 million horses and 25 billion chickens, contributing significantly to the global economy (World Economic Forum, 2019; Food and Agricultural Organization of the United Nations, 2021).

Consider the scale of this integration: The equine veterinary services market is projected to reach $15.9 billion globally by 2027 (Equine Veterinary Services Market, 2022). In Canada, livestock farms allocate around $21 billion in operating expenses annually, with up to 5% dedicated to veterinary services (Lachapelle, 2014). Similarly, American livestock farms invest $357 billion yearly in operating costs, allocating up to 8% to veterinary services (U.S. Department of Agriculture, 2019).

Companion animals also constitute a substantial segment: Approximately, 35% of households in Canada and the United States have dogs, and 38% have cats (American Veterinary Medical Association [AVMA], 2017, 2018a; Canadian Animal Health Institute, 2022). Globally, approximately 471 million dogs and 373 million cats are kept as pets (Nestle, 2019). The US pet industry alone spends approximately $35.9 billion annually on veterinary care and product sales (American Pet Products Association, 2022).

Circadian medicine offers considerable health and welfare opportunities for both agricultural and companion animals. As we explore new frontiers, consider also the rapid expansion of the seafood industry and aquaculture to meet growing human nutrition demands. In 2017, seafood production exceeded 180 million tons from over 400 aquatic species and is expected to increase by an additional 100 million tons by 2050, mainly from cultured species (Stentiford et al., 2022). However, health issues pose a significant constraint to sustainable aquaculture development, costing the global industry over $6 billion annually (World Bank, 2014). In sectors like shrimp farming, infectious diseases alone cause over 40% of production loss (Israngkura, 2002).

**Figure 1.**
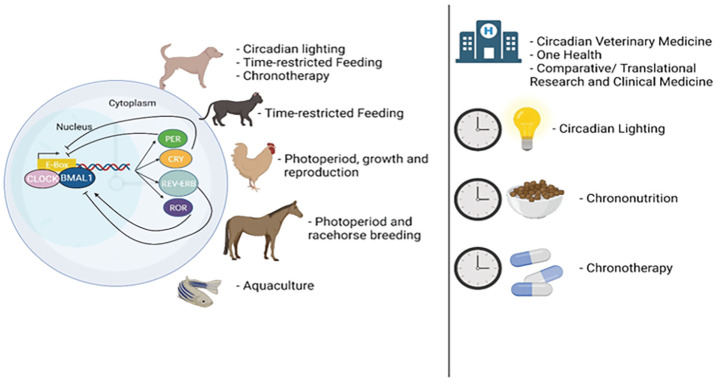
The emergence of circadian medicine for non-human animals has arrived. The circadian mechanism involves a complex interplay of genes and proteins operating in a feedback loop, cycling approximately every 24 h. The factors exhibit coordinated fluctuations, governing both the physiologic and behavioral rhythms in animals. Circadian medicine, a novel therapeutic field, leverages the body’s circadian biology to optimize health, enhance welfare, and modulate responses to diseases. Innovative strategies demonstrating promise for dogs include circadian lighting, time-restricted feeding, and chronotherapy. Time-restricted eating also holds potential for reducing obesity and associated health issues in cats. Manipulating the photoperiod has shown applications in chickens to enhance growth and reproduction. Photoperiod considerations have also shown proven benefits for the racehorse breeding industry. Moreover, considerations around photoperiod manipulation have also proven beneficial for aquaculture and food safety for humans. These applications of circadian medicine notably intersect with Veterinary Medicine’s new frontiers, One Health initiatives, and Comparative/Translational Research and Medicine, bridging the gap between non-human animal and human health. Notably, some of the most promising circadian medicine strategies as discussed in this review include circadian lighting, chrononutrition, and chronotherapy, each presenting considerable potential to extend the lifespan and improve the well-being of non-human animals, transcending benefits solely for humans.

Introducing innovative medical strategies like circadian medicine holds the potential to collectively improve animal health and welfare across various species and sectors. The time has arrived for circadian medicine in non-human animals, offering a compelling array of crucial health applications (summarized in Figure 1, and detailed below).

## Agricultural animals—horses

### Circadian Rhythms in Horses

Since their domestication over 6000 years ago, horses have been an important part of human life and history, serving vital roles in agriculture, transportation, war, sport, and companionship. However, the domestication of horses has considerably altered their exposure to environmental cues that serve to entrain circadian rhythms of physiology and behavior. From highly social, migratory herd animals exposed to natural photoperiods and continuous grazing, today’s equine athletes are placed within confined indoor housing, often in isolation, with regimented feeding, exercise, and social interactions.

### Evidence of Equine Peripheral Cell Clocks

Like most mammals that have evolved on our rotating planet, endogenous circadian rhythms that are synchronized to the environmental L:D cycle govern 24-h physiology in the horse. Initial temporal characterization of circadian clock gene expression patterns in the horse comprised cell culture experiments whereby a media change to high nutrient concentrations was used as a clock resetting signal (mimicking a feeding cue; Balsalobre et al., 1998) and revealed the robust circadian oscillations of equine clock genes in a fibroblast cell line for the first time (Murphy et al., 2006). This work permitted subsequent assessment and confirmation of peripheral clocks in equine adipose tissue, blood cells, skeletal muscle, and hair follicles (Murphy et al., 2007b; Martin et al., 2010; Watts et al., 2012). The finding of robust 24-h rhythms in clock gene expression in hair follicle cells continues to serve as a useful non-invasive method of assessing the circadian system in this species (Watts et al., 2012; Murphy et al., 2021), with potential for use in other species relevant to veterinary medicine.

### Circadian Regulation of Equine Activity

Diurnal variation in equine locomotor activity was previously reported (Berger et al., 1999; Piccione et al., 2002a; Bertolucci et al., 2008), and its circadian component was subsequently confirmed (Martin et al., 2010). A series of experiments investigating activity patterns and skeletal muscle gene expression in horses maintained under three conditions—(1) at pasture as a herd, (2) individually stabled under a normal L:D cycle, and (3) individually stabled in continuous darkness (D:D; a condition necessary to unmask the true endogenous nature of a circadian rhythm)—revealed the diurnal (day-active) nature of this species and confirmed that activity rhythms demonstrated circadian periodicities. Horses were fitted with halter-mounted actigraphy-based monitors that recorded a digitally integrated measure of motor activity and light exposure at 1-min epochs and were sensitive to 0.05 g pressure/movement in any direction (Actiwatch-L, Respironics). These devices were previously used in human studies (Baehr et al., 2003). Ultradian (<24 h) bouts of activity were found to be primarily confined to daylight hours when horses were stabled and higher mean activity counts were only observed during the subjective day under the D:D condition (Martin et al., 2010). This emphasizes the importance of eliminating environmental and management time cues when experimentally determining the endogenous nature of physiological or behavioral rhythms in the horse and other species. During this study, the existence of an oscillating peripheral clock in skeletal muscle was simultaneously confirmed by observations of robust cycling of clock gene transcripts in time series biopsies collected under D:D. It was hypothesized that the molecular clock in muscle tissue regulated the expression of genes involved in muscle hypertrophy, myogenesis, and mitochondrial respiration, pointing to a potential circadian variation in equine performance capacity (Martin et al., 2010). The study served to highlight how equine circadian rhythms can be strengthened, or unmasked when exposed to human management regimes that limit the horse’s natural social entrainment and grazing activity cues. The human tendency to encourage diurnal behavior among domesticated animals has also been noted in dogs, who are naturally crepuscular (active at dawn and dusk), but exhibit diurnal activity patterns through human interactions (Wells, 2017).

Subsequent research evaluating the impact of regular morning exercise on muscle gene expression in horses convincingly showed that time of exercise acts as an important secondary synchronizer of equine skeletal muscle physiology (Murphy et al., 2014a). By exposing previously sedentary Thoroughbreds to an 8-week low-intensity exercise regime, involving 40-60 min each morning on a horse exerciser at walk-trot speeds, a significant shift in the expression pattern of exercise-relevant genes in skeletal muscle was recorded. A circadian rhythm in myogenic differentiation 1 (MYOD1), myogenic factor 6 (MYF6), uncoupling protein 3 (UCP3), and pyruvate dehydrogenase 4 (PDK4) either appeared (from having recorded no 24-h variation before the exercise regime) or was significantly strengthened, and peak expression times shifted relative to the timing of morning exercise (Murphy et al., 2014a). Myogenic regulatory factors MYOD1 and MYF6 contribute to muscle hypertrophy (Psilander et al., 2003) and myogenesis (Montarras et al., 1991). UCP3 is an antioxidant defense mechanism in mitochondria to protect against damaging reactive oxygen species during oxidative stress (MacLellan et al., 2005; Jiang et al., 2009). PDK4 regulates the entry of carbohydrate-derived fuel into muscle mitochondria for oxidation (Pilegaard and Neufer, 2004), increasing substrate availability and production of adenosine triphosphate (Andrews et al., 1998). Given the obvious shift in expression patterns of the aforementioned gene transcripts, the authors concluded that the metabolic capacity of equine skeletal muscle is influenced by a scheduled exercise program (Murphy et al., 2014a). This suggests the potential benefit of aligning training and competition times for optimal performance in the equine athlete, aligning with findings from studies of human athletes (Hill et al., 1989; Hill, 1996; Okamoto et al., 2013). Conversely, mismatches in timing, commonly seen in racehorse training and competition schedules, may increase the risk of musculoskeletal injury.

### Circadian Regulation of Equine Immune Function

A complex bi-directional relationship exists between the immune system and the circadian system in that almost all immune parameters undergo circadian regulation (Scheiermann et al., 2013), and during an acute inflammatory response, the induction of sickness-type behavior (Soto-Tinoco et al., 2016) is regulated by pro-inflammatory cytokines that induce sleepiness, disrupt circadian output, phase shift circadian rhythms, and alter photic entrainment (Cermakian et al., 2013; Labrecque and Cermakian, 2015). In horses, the pro-inflammatory mediator prostaglandin E2 was shown to upregulate the core clock genes PER2 and BMAL1 in ex vivo cultured polymorphonuclear neutrophils (Murphy et al., 2008). This cell population was thus considered responsible for the synchronous upregulation of clock genes in whole blood observed following endotoxin-induced acute systemic inflammation (Murphy et al., 2007b) and confirmed a role for the circadian clock in neutrophil function during an immune challenge in the horse.

From a health management perspective, another important equine rhythm is that of the horse’s core body temperature. The daily temperature range varies by more than 1 °C (Piccione et al., 2002a, 2002b), with a nadir at the start of the light phase (around dawn) and a peak at approximately 2200 h (Piccione et al., 2002a, 2002b; Murphy et al., 2007a). The clear variation between morning and evening values, as well as individual temperature range differences, is important to take into consideration in daily health assessments of animals, particularly in foals where clear dawn-dusk temperature variations are detectable from 10 days old (Piccione et al., 2002a, 2002b).

Clock gene and cytokine response to an antigenic challenge were shown to vary over the 24-h cycle in the horse (McGlynn et al., 2010). In blood samples collected at 4-h intervals and incubated with lipopolysaccharide, interleukin-6 (IL-6) was highest when blood cells collected during the evening hours were stimulated. As an important immunomodulatory mediator produced primarily by Th2 cells (Diehl and Rincon, 2002), this time-of-day–specific peak in IL-6 expression in response to antigenic challenge led the authors to suggest there may be an optimum time for vaccine administration for priming the immune system and for subsequent disease protection (McGlynn et al., 2010), but this is yet to be experimentally validated.

Similarly, chronopharmacology, the concept of clinical treatment that enhances both effectiveness and tolerance and minimizes the side effects of a drug by determining the best biological time for its administration, is an area of veterinary medicine that warrants extensive future research. Considering the breadth of circadian physiology, it makes sense that the pharmacodynamics and pharmacokinetics of many drugs would also be circadian, and thus drug efficacy and safety profiles may vary with time of day (Dallmann et al., 2014).

### Circadian Rhythms in Equine Reproduction

Many equine reproductive functions exhibit time-of-day peaks suggestive of circadian control. Two-thirds of mares ovulate at night (Witherspoon and Talbot, 1970; Bowman et al., 2007) and data from other species suggest that a circadian clock drives the sensitivity of the ovary to luteinizing hormone (LH; Sellix et al., 2010). The circadian rhythm of oxytocin production by the hypothalamus is thought to govern the onset of myometrial activity at parturition (Roizen et al., 2007), potentially explaining why 86% of mares foal between the hours of dusk and dawn (Rossdale and Short, 1967). It was previously postulated that while activation of the fetal hypothalamic-pituitary-adrenal axis due to cramped conditions decided the day of foaling, the mare’s endogenous regulation of myometrial activity by increased production of oxytocin at night fine-tunes the hour of birth to the period of darkness (Murphy et al., 2019).

Stallions exhibit robust daily rhythms in testosterone production, with peaks in the morning and lowest levels recorded between 1600 and 0000 h (Kirkpatrick et al., 1976). This rhythm is related to the circadian control of adrenal glucocorticoid production (Son et al., 2008) and highlights the importance of considering the time of sampling when evaluating testosterone concentrations during fertility assessments of stallions.

### Circadian Disruption in Horses

#### Jet Lag

The consequences of circadian disruption are well recognized for humans, with jet lag, shift work, and light pollution from screens providing erratic entrainment signals that contribute significantly to many disease conditions (Escobar et al., 2011). The circadian misalignment described as jet lag results from a slow readjustment of physiological and behavioral rhythms that shift with different speeds to a new environmental L:D schedule (Davidson et al., 2009). Horses are traveled extensively for breeding and competition purposes making jet lag effects a relevant concern for equine owners and veterinarians. Significant decreases in reaction times, cardiorespiratory functions, and muscle strength have been reported in human athletes following travel across multiple time zones (Manfredini et al., 1998; Lemmer et al., 2002; Reilly et al., 2005).

A study to evaluate the extent of circadian disruption in horses by mimicking an abrupt time zone change evaluated melatonin and body temperature rhythms following a 6-h shift in the L:D cycle (Murphy et al., 2007a). Serum melatonin and core body temperature rhythms are considered robust markers of the circadian phase in humans, and melatonin has been frequently used to provide reliable estimates of circadian adaptation to phase shifts (Akerstedt et al., 1979; Van Cauter et al., 1998; Boivin and James, 2002). In horses, 24-h melatonin production patterns shifted immediately to the 6-h advanced light schedule, whereas disturbances in the body temperature rhythms persisted for the 11-day post-shift sampling period, with greatest disturbances reported on days 7 and 9.

The surprising finding of rapid resynchronization of the melatonin rhythm led the authors to surmise that the equine circadian system may be more amenable to rapid shifts in the L:D cycle than other species (Murphy et al., 2007a). However, subsequent research has shown that melatonin production is not under circadian control in this species, and instead, its production appears to be directly responsive to the photoperiodic conditions (Murphy et al., 2011; Piccione et al., 2013a). Thus, the duration of considerable disruptions observed during resynchronization of the body temperature rhythm is a more reliable reflection of the time required for the horse’s body clock to re-align with the environmental conditions following a 6-h time shift. Future research should evaluate jet lag effects in horses utilizing alternative markers of the circadian clock. Evaluation of clock gene expression in hair follicle cells (Watts et al., 2012) represents a practical, non-invasive way of doing this and has already been implemented in human studies (Akashi et al., 2010; Takahashi et al., 2018). Mitigating potential performance deficits associated with jet lag should reduce the risk of injury at competition destinations.

#### Inappropriate Light Exposure

Modern management of the equine often requires nighttime interventions to monitor, feed, or medicate, and this necessitates the switching on of lights. Regular exposure to inappropriately timed white light at night is considered the primary disruptor of circadian rhythms for the horse (Murphy, 2019). Interference with the nightly production of the pineal hormone melatonin is thought to contribute strongly to this. As a key mediator of the transmission and maintenance of circadian and circannual messages (Brainard et al., 2001), the 24-h pattern of melatonin production that reflects the environmental L:D cycle acts as an important health regulator by optimizing nighttime rest (Paredes et al., 2007) and immune function (Markus et al., 2007). Exposure to bright light at night very strongly suppresses melatonin secretion in humans and horses (Redlin, 2001; Walsh et al., 2013), disrupts circadian rhythmicity, and can profoundly affect many aspects of mammalian physiology (Bartness and Goldman, 1989; Berson et al., 2002; Bedrosian et al., 2011). In humans, frequent white light exposure at night is associated with an increased risk of developing a variety of serious diseases including certain types of cancer (breast, colon, and prostate), obesity, diabetes, and depression (Stevens and Zhu, 2015).

### Circadian Lighting for Equine Health and Medicine

The concept of circadian lighting as it relates to human health stems from our understanding of the beneficial impact of blue light exposure by day and its chrono-disruptive effect by night, for example (Pauley, 2004; Bedrosian et al., 2011, 2013; Bonmati-Carrion et al., 2014; Fonken and Nelson, 2014). In contrast to blue light, low levels of long-wavelength red light only minimally stimulate ipRGCs as has been shown in rodents (Bedrosian et al., 2011). Whereas 5 lux white light intensities disrupted sleep-wake cycles in rats (Stenvers et al., 2016), <10 lux of red light permit normal activity-rest behavior and metabolic function in rats and mice (Opperhuizen et al., 2017; Zhang et al., 2017b). Of course, nocturnal rodents differ in many ways from diurnal animals including humans. Nevertheless, similarities persist, for example, in a study on horses aimed to determine if red light could be used as an alternative to darkness at night to avoid disturbances to the diurnal pattern of melatonin secretion, no differences were observed in its 24-h pattern and waveform characteristics in horses maintained under L:D compared to light-red photo-schedules (Murphy et al., 2019). The low-intensity red light (5 lux, peak wavelength 625 nm, 0.3 µW/cm^2^/nm) used in the study permitted sufficient visibility to safely carry out routine animal husbandry, collection of rectal temperatures, blood sampling, and blood collection tube labeling—important routine tasks for animal care in breeding facilities or veterinary hospitals. The authors suggested that like rodents, red light at night may be functionally equivalent to total darkness concerning its impact on the equine circadian system, and thereby its use may permit normal maintenance of nocturnal circadian physiology (Murphy et al., 2019).

These studies have important translational implications for care environments. It is well known that unnatural ambient lighting conditions in human intensive care units (ICUs) and experimental rodent studies lead to poor sleep, melatonin suppression, delirium, and other chronobiologically disruptive effects on a large number of important organ systems (Figueroa-Ramos et al., 2009; Boyko et al., 2012; Chan et al., 2012) as well as influencing survival outcome following sepsis in rats (Carlson and Chiu, 2008), and impair cardiac repair in mice following myocardial infarction (Alibhai et al., 2014). Combined with a recent study in human athletes reporting that exposure to short durations of red light at night improved sleep and athletic performance (Zhao et al., 2012), it was postulated that the benefits of consistent exposure to long-wavelength light at night in horses may extend beyond a simple means to eliminate unwanted circadian disruption caused by shorter wavelengths (Murphy et al., 2019).

The sensitivity of horses to blue wavelength light was highlighted by a study demonstrating the effectiveness of levels as low as 10 lux at acutely suppressing serum melatonin when administered to a single eye (Walsh et al., 2013). A mobile headpiece that delivers timed blue light to one eye to extend daylight was subsequently developed and has been shown to effectively advance the circannual rhythm of reproductive activity in mares (Murphy et al., 2014b) and influence gestation length (Nolan et al., 2017), foal maturity (Lutzer et al., 2022), and coat condition in horses (O’Brien et al., 2020).

By combining the findings of the preceding research studies, the same researchers developed a customized light-emitting diode (LED) lighting system for equine housing that comprised timed blue-enriched daytime polychromatic white light (peak wavelength 450 nm and 0.8 µW/cm^2^/nm) and nighttime monochromatic red light (625 nm, 0.3 µW/cm^2^/nm) and an investigation to determine its impact on peripheral clock gene rhythmicity compared to standard lighting practices in a racehorse training yard undertaken (Murphy et al., 2021; Collery et al., 2023). After 20 weeks of exposure to the customized LED stable lighting, 24-h clock gene expression of PER2 and NR1D2 was found to be rhythmic in hair follicle cells (as determined by cosinor analyses), whereas no rhythmicity was apparent in samples collected from horses maintained in stables with incandescent lights that were used at will by the staff. The strengthened oscillatory expression of core clock genes observed in hair follicle cells of horses exposed to blue-enriched daytime polychromatic light and nighttime red light leads the authors to strongly suggest that circadian synchrony is improved throughout body tissues under these lighting conditions (Murphy et al., 2021). A second important implication of this research was that standard management regimes for stabled horses disrupt circadian cohesion and potentially contribute to sub-optimal health and compromised immunity (Collery et al., 2023).

As previously highlighted, the provision of lighting environments that support internal circadian cohesion, maintaining synchrony between the SCN and peripheral oscillators, could have widespread benefits for equine health and performance (Murphy, 2019). Future investigations should aim to evaluate the impact of custom LED lighting regimes, such as those previously described (Collery et al., 2023), on the health and behavioral parameters of the horse. Indeed, preliminary research indicates that exposure to blue LED light may improve some symptoms of Pituitary Pars Intermedia Dysfunction (Miller et al., 2022).

## Agricultural animals—chickens

### Circadian Rhythms in Chickens

Birds, including chickens, have a highly complex and diversified circadian system. While the retinal circadian clock was initially identified in the African clawed frog, *Xenopus laevis* (Besharse and Iuvone, 1983) has been conserved across the avian species (McMillan et al., 1975; Ebihara et al., 1984; Barrett and Underwood, 1991), and additional self-sustaining circadian oscillators have been identified in the pineal gland and hypothalamus of birds (Ebihara et al., 1984; Zatz and Mullen, 1988; Okano et al., 1994; Natesan et al., 2002). Notably, in birds, the avian retina is not indispensable for circadian entrainment (Menaker, 1968; Menaker et al., 1970). Enucleated house sparrows maintain their biological rhythms under standard day length periods due to these extra-retinal oscillators. This is supported by observations that birds develop arrhythmicity when light cannot penetrate the skull, despite intact eyes, demonstrating the necessity of encephalic circadian oscillators for entrainment (Menaker et al., 1970; Foster and Follett, 1985).

Of note, avian photoreceptors also reside in these three photoreceptive organs, including rhodopsin and melanopsin (OPN4) in the retina; pinopsin, OPN4, and vertebrate-ancient opsin (VA-Opsin) in the pineal gland; and VA-Opsin, OPN4, and neuropsin (OPN5) in the hypothalamus (Foster et al., 1985, 1994; Chaurasia et al., 2005; Halford et al., 2009; Kang et al., 2010; Nakane and Yoshimura, 2010; Davies et al., 2012; Ohuchi et al., 2012). These photoreceptors in encephalic tissues sustain biological rhythms by capturing and transducing photons through the skull, relaying this information to peripheral circadian oscillators (Ebihara and Kawamura, 1981; Takahashi and Menaker, 1982; Cassone and Moore, 1987).

In birds, both the retina and pineal gland detect changing day lengths (Gaston and Menaker, 1968; Binkley et al., 1971; Ebihara and Kawamura, 1981; Lu and Cassone, 1993; Wang et al., 2012) through elevations in tryptophan hydroxylase (Chong et al., 1998), arylalkylamine *N*-acetyltransferase (AANAT; Bernard et al., 1997), and hydroxyindole-O-methyltransferase during the dark phase (Hamm and Menaker, 1980; Thomas and Iuvone, 1991), resulting in increased melatonin production and release from the pineal gland (Deguchi, 1979; Hamm and Menaker, 1980; Takahashi et al., 1980). Melatonin production decreases in response to light stimulation, demonstrating a circadian rhythm under 24-h L:D cycles. However, arrhythmic responses have been observed under continuous 24-h light or 24-h dark photoperiods, with melatonin rhythmicity absent under both conditions (Saito et al., 2005; Ozkan et al., 2012; Honda et al., 2017; Ma et al., 2019). These studies establish light stimuli as a primary zeitgeber in avian species.

Earlier studies in pigeons and house sparrows also investigated feeding schedules as an additional possible zeitgeber (Phillips et al., 1993; Rashotte and Stephan, 1996). However, little progress has been made in this area. Food-entrainable oscillators can be desynchronized from the photic response. This has been demonstrated as an earlier onset of photophase alters the rise of core body temperature and oxygen consumption, typically identified as anticipatory feeding behaviors, with the birds gradually re-entraining their feeding schedule within days (Rashotte and Stephan, 1996). Feeding regimens serve as a weak zeitgeber (Hau and Gwinner, 1992). Desynchronized birds can demonstrate “masking” behavior and adjust their feeding patterns accordingly (Hau and Gwinner, 1996). Honda and colleagues (2017) observed constant feed intake of male broiler chicks throughout 24 h of continuous light and increased feed intake before and following the dark period of a 12L:12D photoperiod. However, despite differences in feed intake, the expression of an appetite-stimulating peptide, neuropeptide Y, and an appetite-suppressing peptide, termed pro-opiomelanocortin, did not differ between these photoperiods (Honda et al., 2017) This suggests minimal interaction between the circadian rhythm and the melanocortin system in chickens, warranting further investigation into the mechanisms behind the control of feed intake and photoperiods.

### Current Industry Practices

Over the past century, there has been a substantial increase in the production capacity of meat (broilers) and table eggs (laying hens), shifting production from a dual-purpose backyard flock model to larger scale farms with specific, divergent breeding goals (Lawler, 2012). In these larger indoor systems, producers have gained greater control over environmental factors like temperature, humidity, ventilation, and lighting. This control allows year-round production in temperate zones.

Producers often adjust the length of photoperiodic exposure to benefit the health of broilers (as reviewed by Classen et al., 1991) and to enhance the reproductive efficiency of laying hens and broiler breeders (as reviewed by Sharp, 1993).

Given that photoperiod is the primary zeitgeber in poultry, various lighting programs have been implemented over the years, each with its advantages and challenges. Industry standards differ for broilers and laying hens based on breeding objectives. Broilers are typically reared under longer day lengths to allow for extended feed access and to promote growth (Cobb-Vantress, 2020). Meanwhile, to optimize reproductive performance, laying pullets are typically maintained under short-day lengths of less than 10 h, stepping up to 16L:8D at the time of maturation, which occurs at approximately 18 and 22 weeks of age in layers and broiler breeders, respectively (Aviagen, 2016; Lohmann-Tierzucht, 2021).

### Implications of Spectrum Lighting on the Circadian Rhythm

In the commercial context, alongside manipulating the photoperiod, there has been extensive research into using spectrum lighting, especially with the phasing out of inefficient lighting systems such as incandescent bulbs and the introduction of LEDs. It is largely understood that hens housed under red wavelengths exhibit enhanced reproductive capacity (Mobarkey et al., 2010; Min et al., 2012; Hassan et al., 2013; Baxter et al., 2014), while those under green wavelengths show increased skeletal muscle cell proliferation leading to improved growth (Halevy et al., 1998; Rozenboim et al., 2004). Recent research indicates that these wavelengths directly impact the circadian rhythm.

Under green light, gene expression in the positive arm of the circadian mechanism (*Clock* and *Bmal1*) has been shown to increase in expression (Jiang et al., 2016; Cao et al., 2017; Jiang et al., 2020; Yang et al., 2020), and activate the melatonin regulatory factor AANAT (Cao et al., 2017; Jiang et al., 2020; Yang et al., 2020), whereas gene expression in the negative arm (*Per* and *Cry*) are downregulated (Jiang et al., 2016; Yang et al., 2020). This leads to a concomitant elevation in melatonin production under green wavelengths (Jiang et al., 2016; Cao et al., 2017; Bian et al., 2020; Jiang et al., 2020; Yang et al., 2020). In contrast, an opposite effect has been observed under red wavelengths, with gene expression in the negative arm upregulated (Cao et al., 2017; Yang et al., 2020) and those in the positive arm downregulated in the hypothalamus (Cao et al., 2017; Jiang et al., 2020; Yang et al., 2020). Interestingly, *Bmal1* seems elevated in the pituitary gland of female birds only, although no further studies investigating this have been conducted to date (Wang et al., 2015).

Blue light, the shortest wavelength, negatively affects gene expression in both the positive and negative arms of the circadian mechanism (Yang et al., 2020), and inhibits melatonin secretion (West et al., 2011). Surprisingly, maintaining male broiler chicks under a 24-h lighting program consisting of 12 h of white light and 12 h of blue light sustains circadian gene expression (Honda et al., 2017). Presumably, birds perceive blue light in a similar manner to scotophase when coupled with other spectrums with longer wavelengths. The implications of these altered circadian patterns will be further explored concerning growth and egg-laying applications.

### Photoperiodic Control of Reproduction in Laying Hens and Broiler Breeders

Chickens, being seasonal breeders, heavily rely on their circadian and circannual systems to regulate their reproductive processes. Short and long photoperiods play roles in inhibiting and stimulating the hypothalamic-pituitary-gonadal (HPG) axis, respectively (Tsutsui et al., 2000; Bentley et al., 2003; Ubuka et al., 2005).

To maintain an immature state, pullets are exposed to short-day conditions (less than 10 h of light), triggering the release of melatonin (Ubuka et al., 2005). Melatonin then upregulates the expression of gonadotropin-inhibitory hormone (GnIH). GnIH, upon binding to its receptor, directly inhibits the release of gonadotropin-releasing hormone (GnRH) and gonadotropins (like follicle-stimulating hormone and LH) effectively suppressing the HPG axis.

During photo-stimulation, reducing the duration of darkness reduces melatonin production, leading to a decrease in GnIH expression. The mediobasal hypothalamus (MBH) containing a molecular circadian mechanism (Yasuo et al., 2003) then integrates the changes in photic information to stimulate thyrotrope cells in the pars tuberalis of the pituitary gland to produce thyroid-stimulating hormone (TSH). Increased TSH levels prompt tanycytes on the base of the third ventricle to upregulate type 2 deiodinase (Dio2), an enzyme upregulated under longer days (Yoshimura et al., 2003). Dio2 facilitates the conversion of thyroxine to triiodothyronine (T_3;_ Bernal, 2002; Nakao et al., 2008). Elevated levels of T_3_ within the MBH act on the median eminence, allowing GnRH nerve terminals to interact with the basal lamina and release this stimulatory neuropeptide, activating the remainder of the HPG axis (Prevot et al., 1999; Yamamura et al., 2004; Yamamura et al., 2006).

While this downstream process is well established, the receptor responsible for responding to light and signaling this cascade of events remains unclear. Current hypotheses suggest a single “breeding opsin” initiates HPG axis activation, with potential photoreceptors including VA-Opsin (Hankins et al., 2008; Halford et al., 2009; Garcia-Fernandez et al., 2015), OPN4 (Foster et al., 1987; Bailey and Cassone, 2005; Hankins et al., 2008), and OPN5 (Tarttelin et al., 2003; Halford et al., 2009; Nakane and Yoshimura, 2010) located in deep brain regions. Studies on their involvement in seasonal responses have been recently reviewed (Hanlon et al., 2020).

As hens reach sexual maturity, their reproductive tract synchronizes with circadian oscillators and genes related to the circadian mechanism to regulate the ovulatory cycle’s timing (Fahrenkrug et al., 2006; Karman and Tischkau, 2006; Nakao et al., 2007; Yoshikawa et al., 2009). This cycle can be entrained to photoperiods between 21 and 30 h, varying by species. In domesticated laying hens exposed to a 16-h light and 8-h dark cycle (16L:8D), this process slightly exceeds a 24-h rhythmic cycle. Notably, there are two separate rhythms—one governing ovulation and the other controlling follicle growth and maturation. When these align, it leads to the laying of eggs on consecutive days, termed a clutch (Bahr and Johnson, 1984). These eggs are laid within a 6-10–h window known as the “open period.” If these rhythms become desynchronized, a pause day without egg-laying resets the cycles (Etches et al., 1984).

The LH surge before ovulation acts as a zeitgeber, influenced by circadian mechanism genes (Tischkau et al., 2011). LH acts via steroidogenic acute regulatory protein and BMAL1 (Nakao et al., 2007), increasing progesterone just before ovulation (Tischkau et al., 2011). The importance of this ovarian circadian system is evident in ovariectomized hens, where core body temperature becomes arrhythmic (Underwood et al., 1997; Zivkovic et al., 2000), and in hens under 24-h light cycles where hormonal profiles maintain ovulatory cycles (Kadono et al., 1981). Interestingly, only the most mature follicles (F1-F3) exhibit rhythmic responses when exposed to LH, while less mature preovulatory follicles do not display rhythmic expression of these circadian genes (Zhang et al., 2017a). This suggests that rhythmic interaction develops as the follicle matures. The central circadian system likely controls this rhythm. For instance, hens under 16L:8D supplemented with exogenous melatonin will lay eggs with significantly larger yolks due to disrupted timing and extended rapid growth for lipid deposition (Taylor et al., 2013). A key message is manipulating photoperiods can influence the egg weight and internal components, potentially impacting industry standards.

### Lighting Applications for Reproduction

Studies exploring light duration and spectrum aimed to improve reproductive capacity have revealed important findings. It was observed that longer day lengths can stimulate the reproductive axis, leading to investigations into continuous 24-h lighting. However, when mature hens were constantly exposed to light, it disrupted the synchronization of critical follicular maturation, LH surges, ovulation, and oviposition (Dawson and Goldsmith, 1997). Consequently, the overall production rate through the cycle decreased (Callenbach et al., 1944; Wilson et al., 1956).

Efforts were made to address these challenges through ephemeral lighting programs, extending the total hours of light beyond 24 h to align with natural ovulatory rhythms (Byerly and Moore, 1941). Unfortunately, this was unsuccessful, as eggshell quality and weight improved at the expense of the production rate (Leeson and Summers, 1988). The variation of the open window period within individuals in a flock likely contributed to these unfavorable results.

Overall, the current industry practices, using 24-h L:D cycles for photo-stimulation, seem to strike a balance by leveraging the physiological benefits while maintaining circadian rhythmicity.

When considering spectrum lighting for birds, it is first important to acknowledge that birds have extra-retinal photoreceptors, present in places like the pineal gland and hypothalamus. The penetrability of light through bone and nervous tissue is dependent on wavelength. Remarkably, hens maintained under red wavelengths demonstrate earlier sexual maturation and greater cumulative production (Min et al., 2012; Hassan et al., 2013), regardless of retinal stimulation (Baxter et al., 2014), emphasizing the significance of extra-retinal deep brain photoreception. Interestingly, this effect is achieved by downregulating the positive arm of the circadian genes and AANAT, which lowers melatonin production (Jiang et al., 2016; Bian et al., 2020; Yang et al., 2020). While this suggested that red light may synchronize reproductive processes, further research is needed to fully understand its role in hen circadian biology.

Conversely, green light, known to increase melatonin production, delays the onset of egg-laying. This aligns with how melatonin interacts with GnIH in the hypothalamus (Min et al., 2012; Hassan et al., 2013; Baxter et al., 2014). The decreased production rate observed with green light aligns with melatonin’s ability to delay the initiation of each egg-laying cycle (Greives et al., 2012). Interestingly, the delay caused by green light is more pronounced when the retina remains intact compared to hens with retinal degeneration. This suggests that the retina plays a significant role in melatonin production specifically under green light conditions (Baxter et al., 2014).

### Photoperiodic Control of Bone Growth and Development

Early studies in layers revealed the vital role of melatonin in maintaining skeletal health and preventing bone disorders like scoliosis (Machida et al., 1995; Wang et al., 1998). While a pinealectomy contributes to anywhere between 52% and 100% of birds developing scoliosis, melatonin treatment can reduce the severity or even entirely prevent this disease (Machida et al., 1995). Further investigation has revealed that melatonin stimulates osteoblastic proliferation and differentiation—the cells responsible for bone formation (Nakade et al., 1999; Cardinali et al., 2003; Park et al., 2011). Simultaneously, it inhibits the activation and formation of osteoclasts, which break down bone tissue (Koyama et al., 2002). Melatonin also supports the production of type I collagen, a critical component of bone structure (Nakade et al., 1999). Without melatonin, the normal bone growth process at the epiphyseal plate—a critical area for bone development—is disrupted (Aota et al., 2013). These findings emphasize how adjusting the length of the dark period can greatly impact the growth and development of bones, a phenomenon that has been especially studied in broiler chicks and layer hens.

### Broiler Leg Health

Broilers have been intensively bred for rapid growth and efficient feed consumption, usually kept under prolonged 18-23–h periods of light to encourage extensive feeding (Cobb-Vantress, 2020). However, while extended photoperiods promote increased feed intake, short scotophases demonstrate an abolishment of circadian rhythmicity, decreasing locomotor activity. In combination, this results in rapid weight gain that in turn leads to the development of leg abnormalities, including but not limited to lameness and tibial dyschondroplasia (Classen and Riddell, 1989; Sorensen et al., 1999), impacting their movement, ability to access food and water, and often ending with on-farm culling. Thus, this is a major economic and welfare concern within the industry.

Broilers with leg weakness preferentially select feed supplemented with an analgesic agent (McGeown et al., 1999; Danbury et al., 2000), indicating considerable discomfort associated with these disorders (Sorensen et al., 1999). However, shortening the photoperiod has been proven effective in reducing the incidence of leg abnormalities and is coincident with the maintenance of rhythmic melatonin production (Taylor et al., 2013; van der Pol et al., 2017; van der Pol et al., 2019).

### Laying Hen Bone and Shell Development

Laying hens require 2.2 g of calcium for deposition on the shell of each egg produced, equating to about 10% of their total calcium content on a 24-h basis (Bouvarel et al., 2011). Thus, calcium homeostasis is critical to their health, welfare, and the quality of the consumer product. In the case of breeder layers, this shell quality becomes even more important due to its role in providing calcium to the growing embryo for cartilage formation (Qi et al., 2016; Torres and Korver, 2018). To accommodate the demands of eggshell deposition during scotophase, hens maintain a specialized and readily labile source of bone within the endosteal surface of long bones (Bloom et al., 1941; McCoy et al., 1996), referred to as medullary bone. This bone source provides about 30% of the total required calcium, in addition to the dietary source, to prevent the breakdown of structural bone (Comar and Driggers, 1949; Mueller et al., 1964). However, osteoporosis is a common disease in laying hens resulting in a disorder termed cage layer fatigue. It is highly prevalent and a major cause of poor health and welfare (Whitehead and Fleming, 2000).

Calcium homeostasis appears to be partially regulated by the circadian mechanism. The timing of calcium consumption can entrain circadian oscillators, acting as a potential zeitgeber for the circadian oscillatory mechanism in the kidney of hens (Damiola et al., 2000; Lin et al., 2018). When the same levels of calcium are fed in the morning and evening, the circadian genes in the intestinal jejunum and in the kidney of laying hens exhibit normal daily rhythmicity. However, if the calcium provided is higher in the morning and lower in the evening, these circadian genes become arrhythmic (Lin et al., 2018). In addition, serum calcium levels display a circadian rhythm (Sloan et al., 1974), which can be altered through photoperiod manipulations (Pablos et al., 1995). These findings suggest that varying photoperiods can mechanistically impact the timing of eggshell formation.

In addition to calcium intake, calcium deposition and transport of calcium to the shell gland also occur rhythmically due to the circadian expression of 1,25-dihydroxy vitamin D_3_ (1,25[OH]_2_D_3_; Abe et al., 1979; Frost and Roland, 1991). Initially, it was thought that the circadian rhythm of the ovulatory cycle’s sex hormones controlled 1,25(OH)_2_D_3_ rhythms (Peterson and Common, 1972; Abe et al., 1979), but studies disproved this when hens laid shell-less eggs despite normal sex hormone patterns when 1,25(OH)_2_D_3_ lost its rhythmicity (Nys et al., 1986).

### Lighting Applications for Improving Bone Growth and Development

Recent studies have examined the effects of photoperiods during incubation on early bone development. Continuous light exposure during embryonic growth negatively impacts melatonin production, and also results in weaker bones with higher rates of tibial dyschondroplasia. Conversely, using continuous dark conditions typically used in hatcheries does not cause such detrimental effects. Chicks incubated in D:D often show earlier ossification in the tibia and femur. However, using a 12L:12D dark cycle during incubation has shown additional benefits, like increased osteoblast activity by embryonic day 13 (van der Pol et al., 2019). This suggests that L:D cycles promoting circadian patterns might positively influence embryonic growth and extend benefits to the chick. While current research is focusing on spectrum lighting during this period, no data on its impact on circadian rhythms are available.

Given that melatonin, beneficial for bone development, increases under green light, it is hypothesized that using this spectrum during incubation and early growth be advantageous. While most studies focus on broiler chicks, similar improvements in bone development might be seen in laying hens, potentially reducing the incidence of osteoporosis later in their lifespan. Beyond its bone benefits, melatonin, as an anti-inflammatory agent, is suppressed by inflammatory substances that decrease AANAT and clock gene expression (Majewski et al., 2005a, 2005b; Carrillo-Vico et al., 2013). The prevalent use of continuous light in the industry might elevate inflammatory markers, negatively impacting animal health (Majewski et al., 2005a; Shini et al., 2010). Thus, considering the light spectrum environment could potentially enhance animals’ immune status and overall health in the industry.

## Companion animals—dogs

### Circadian Rhythms and Effects of Rhythm Disruption in Dogs

Dogs are the descendants of wolves. Their domestication by humans is thought to have occurred at least 15,000 years ago, and possibly much earlier as they formed beneficial relationships with humans. In the wild, wolves typically have a diurnal activity pattern where they are most active during the day and rest at night. However, there is some controversy in the literature regarding circadian activity patterns, as this can vary depending on the availability of prey, and in the winter months, they may become even more active at night (Theuerkauf, 2009; Eriksen et al., 2011, 2022; Merrill and Mech, 2023). Dogs can also tend toward crepuscular behavior, but that is modified by being with humans such that domesticated dog breeds typically exhibit only diurnal locomotor and activity-rest patterns, following similar patterns as their human owners (Nishino et al., 1997; Zanghi et al., 2012; Wells, 2017; Jean-Joseph et al., 2022). Experimental and clinical studies reveal robust diurnal circadian rhythms in domesticated canine heart rate (Ashkar, 1979; Matsunaga et al., 2001), blood pressure (Ashkar, 1979), body temperature (Refinetti and Piccione, 2003), respiratory rate (Ashkar, 1979), bone metabolism (Liesegang et al., 1999), and heat dissipation (Besch and Woods, 1977), similar to other diurnal mammals. Somewhat surprisingly, daily rhythms in endocrine hormones such as adrenocorticotropic hormone, cortisol, and thyroxine remain controversial in dogs as results differ between studies (Kemppainen and Sartin, 1984; Palazzolo and Quadri, 1987), even though daily rhythmicity in these hormones is prevalent in humans and other diurnal mammals (Gamble et al., 2014). Understanding circadian rhythms in dogs is important for healthy canine physiology and well-being.

Disruption of a dog’s circadian rhythm may have detrimental effects on their physiology, behavior, and overall health. Unfortunately, in our modern society, circadian disruption is often unavoidable due to prolonged exposure to artificial lighting, which extends our daylight hours (photoperiods). This affects not just humans, but our companion animals as well. One consequence for dogs is that disrupting daily rhythms may increase the risk for metabolic disorders. For example, a study demonstrated that just one night of disruption in dogs significantly diminished insulin sensitivity (Brouwer et al., 2020). However, it is worth noting that study achieved disruption through constant contact rather than changes in environmental lighting, leaving further research necessary to fully understand the effects of circadian disruption on metabolism in dogs.

Another perspective on how circadian disruption affects the physiology of dogs can be examined through the lens of canonical light entrainment pathways. Dogs possess the ipRGCs responsible for light detection and SCN entrainment (Yeh et al., 2017). They are sensitive to light intensity too: Low luminous intensity (<50 lux) at night promotes sleep, while high illumination (>1600 lux) at night disrupts this behavior (Fukuzawa and Nakazato, 2015). This understanding matters because studies in humans and other daytime-active animals show that low nighttime light fosters pineal melatonin production, while increased nighttime light suppresses melatonin, potentially disrupting body processes (Gooley et al., 2011; Arendt, 2019). Although our understanding of pineal melatonin in dogs is limited, their peripheral blood shows a rhythmic pattern, peaking at night (Stankov et al., 1994), similar to what is observed in humans.

### Circadian Medicine: Using Light Timing to Benefit Health in Dogs

Various studies, both in experimental animals and humans, emphasize the importance of daily rhythms for maintaining health across numerous body processes. These rhythms influence things like autonomic nervous system bias (Reitz et al., 2022), blood vessels (Kroetsch et al., 2022), autophagy (Rabinovich-Nikitin et al., 2021), inflammatory responses (Alibhai et al., 2015; Khaper et al., 2018; Rabinovich-Nikitin et al., 2019; Aziz et al., 2021), heart health (Young et al., 2014; Martino and Young, 2015; Mia et al., 2020), diurnal gene and protein rhythms (Martino et al., 2004, 2007a, 2007b; Chalmers et al., 2008a, 2008b; Tsimakouridze et al., 2012; Alibhai et al., 2014; Podobed et al., 2014a, 2014b; Bennardo et al., 2016; Hughes et al., 2017), and numerous other processes (e.g., see Martino and Sole, 2009; Scheer et al., 2009; Sole and Martino, 2009; Kinlein et al., 2015; Labrecque and Cermakian, 2015; Martino and Young, 2015; Reutrakul and Knutson, 2015; Morris et al., 2016; Martino and Young, 2017; Reutrakul and Van Cauter, 2018; Thosar et al., 2018; Haspel et al., 2020; Boivin et al., 2022; McEwen and Karatsoreos, 2022).

Disruptions to normal L:D cycles have been linked to disease development and can hinder the body’s repair processes. In dogs, excessive nighttime light exposure might affect their physiology similarly to what is seen in other animals and humans, potentially impacting diseases like heart issues and cancer, common in both dogs and humans.

From a clinical perspective, these studies indicate that pet owners acknowledge and prioritize their dogs’ circadian rhythms. Establishing a consistent daily routine can actively contribute to their pet’s overall well-being and good health.

### Circadian Medicine: Using Time-restricted Eating to Address Obesity and Related Comorbidities in Dogs

Maintaining circadian rhythms is crucial for overall health, and the emerging field of circadian medicine offers potential therapeutic strategies for various conditions, including obesity in dogs. In the United States, the prevalence of overweight and obese dogs, as assessed by veterinarians, is reported to be 34% and 5%, respectively (AVMA, 2018a). These conditions are associated with increased risk of comorbidities and reduced lifespan (Kealy et al., 2002; Adams et al., 2015). Factors such as aging, neutering, reduced exercise, and inappropriate feeding practices contribute to weight gain in dogs (Perry et al., 2020).

One promising approach for treating obesity is time-restricted eating (TRE; Vollmers et al., 2009; Hatori et al., 2012; Melkani and Panda, 2017), which has gained attention in human circadian medicine and for cardiometabolic diseases (Mattson et al., 2014; Chaix et al., 2019; Quist et al., 2020; Wilkinson et al., 2020; Crose et al., 2021; Prasad et al., 2021). TRE involves limiting the daily time window for food consumption. Dogs, like other mammals, have sensitive circadian systems that respond to meal timing. Studies on dogs show hormone levels like ghrelin peak before feeding and drop afterward, while leptin peaks 5-8 h post-meal (Ishioka et al., 2005; Yokoyama et al., 2005), suggesting meal timing influences these hormone peaks (Ishioka et al., 2005; Yokoyama et al., 2005).

However, understanding the effects of TRE in dogs is complex due to their classification as opportunistic carnivores. Wolves, for instance, can eat up to 22% of their body weight in a single meal (Stahler et al., 2006), and dogs can survive for extended periods without food (Howe et al., 2012), indicating resistance to prolonged food deprivation. In addition, wild canids experience seasonal variations in food availability, suggesting they can respond to changes in meal frequency. Furthermore, pet owners often align their dog’s feeding schedules with their meals, or that of other companion animals (Laflamme et al., 2008).

Despite these complexities, TRE holds potential health benefits for companion dogs’ health. Studies show that intermittent fasting, along with a high-fat diet, can improve insulin sensitivity and reduce fasting glucose levels compared to daily feeding. Dogs on intermittent fasting with a low-fat, high-carbohydrate diet consumed fewer calories and lost more weight than those on daily feeding or a high-fat diet (Leung et al., 2020). Observational data from more than 20,000 dogs indicate that the once-daily feeding of adult dogs is associated with a reduced risk of various age-related health conditions (Bray et al., 2022). Further research on TRE’s effects in obese dogs, including weight loss, better metabolic health, reduced disease risk, increased energy, and improved sleep, is needed.

### Circadian Medicine: Chronotherapy for the Treatment of Cardiovascular Diseases and Cancer in Dogs

Cardiovascular disease is a prevalent and often fatal condition in dogs, with an estimated 7.8 million affected in the United States, representing around 10% of the canine pet population (Lombard et al., 2006; Haggstrom et al., 2008; O’Grady et al., 2008; Atkins et al., 2009; O’Grady et al., 2009). Conditions like congestive heart failure (Guglielmini, 2003), mainly due to myxomatous mitral valve disease (Borgarelli and Buchanan, 2012), impact about 75% of dogs over the age of 16 (Guglielmini, 2003). Dilated cardiomyopathy is also prevalent in various breeds (Dukes-McEwan et al., 2003; McCauley et al., 2020), and especially Doberman Pinschers (O’Grady et al., 2009), and proceeds unremittingly toward heart failure.

Conventional treatments such as angiotensin-converting enzyme inhibitors (ACEIs) or angiotensin receptor blockers (ARBs) have limited success, and cardiovascular disease remains a leading cause of death in dogs. Novel strategies are needed, and recent studies in rodents (Martino and Sole, 2009; Martino et al., 2011; Martino and Young, 2015; Tsimakouridze et al., 2015), and humans (Hermida et al., 2021; Gumz et al., 2023), suggest potential benefits of chronotherapy with ACEIs or ARBs. Chronotherapy aligns treatments with the body’s circadian rhythms and has shown promise in reducing adverse cardiac changes, lowering nighttime blood pressure, and slowing heart failure progression. Considering that ACEIs are commonly used for heart disease in dogs (Lefebvre et al., 2007; O’Grady et al., 2008), investigating chronotherapy’s potential benefits in enhancing drug effectiveness is a logical step.

Chronotherapy might also impact cancer treatment, as many widely used medications target products of circadian rhythmic genes (Zhang et al., 2014; Ruben et al., 2019b). Healthy tissues and tumors have differing susceptibility to chemotherapy based on the time of day (e.g., Innominato et al., 2014; Ballesta et al., 2017; Lee et al., 2021). Dogs, like humans, are prone to cancer, with a quarter developing it in their lifetime and nearly half of all dogs over the age of 10 face cancer (AVMA, 2023). Chronotherapy in veterinary cancer care could improve tolerance, and safety, and increase tumor sensitivity to treatment.

On a final note, it is likely also important to consider the feeding patterns of owned dogs when developing chronotherapy. Owners often vary in how frequently they feed their pets, and this frequency can influence the metabolic rhythms in dogs. By aligning scheduled feeding with medication administration, it may be possible to enhance the timed delivery of drugs, which could impact their effectiveness. Research into how drugs interact with the body’s rhythms at different times of the day—pharmacokinetics and pharmacodynamics—is a crucial area to explore (Dallmann et al., 2016). Further investigations are eagerly anticipated to fully uncover the benefits of chronotherapy in veterinary medicine.

## Companion animals—cats

### Circadian Rhythms in Cats

Literature characterizing circadian rhythms in domestic cats is scarce and contradictory. Early reports suggested a lack of rhythmicity in activity and body temperature (Sterman et al., 1965; Hawking et al., 1971); however, research has since demonstrated circadian fluctuations in total sleep time and brain temperature indicating a bimodal pattern of wakefulness at dusk and dawn, supporting the notion of crepuscular rhythms under artificial L:D cycles (Kuwabara et al., 1986). These rhythms are endogenously produced and not merely a response to L:D cycles, as cats were observed to have free-running circadian organization of activity and feeding behavior when kept in D:D and arrhythmicity when kept in constant light (L:L; Randall et al., 1985). A more recent study further demonstrated the bimodal profile of daily rhythms in domestic cat locomotion using automatic recording technologies (Parker et al., 2019). Intriguingly, cats also appear to display similar bimodal circadian rhythms in endocrine hormones including norepinephrine (Reis et al., 1969) and melatonin (Reppert et al., 1982), and in daily blood pressure cycling (Mishina et al., 2006). In contrast, rhythms in plasma aldosterone (Yu and Morris, 1998) are absent, while they are evident in humans and other mammals.

Characterizing the daily rhythms of the domestic cat has proven difficult, as recent studies have demonstrated that cats exhibit different chronotypes according to their housing conditions (Piccione et al., 2013b). In addition, as cats are considered symbionts to humans, their activity and feeding behaviors are affected by human interaction (Randall et al., 1985). Collectively, these studies are consistent with the presence of a bimodal profile of circadian rhythms in cats, with crepuscular peaks associated with twilight. Indeed, cats exposed to 16 h of light and 8 h of dark had greater physical activity and good intake and resting metabolic rate, in contrast to cats exposed to an 8L:16D cycle, suggesting that the length of day could have a significant impact on energy balance although the mechanism by which this occurred was not clear (Kappen et al., 2013). Ultimately, by improving our understanding of feline circadian rhythms, we may be able to further develop nutritional and housing guidelines to support health and recovery from disease.

### Circadian Medicine: Feeding Frequency for the Prevention and Treatment of Obesity and Diabetes in Cats

Similar to dogs, obesity and its associated comorbidities in cat populations are a growing concern worldwide (German, 2006; German et al., 2010; Chandler et al., 2017; AVMA, 2018b). The percentage of overweight and obese cats was recently estimated to be between 22% and 52%, respectively, depending on study parameters and country of origin (Colliard et al., 2009; Rowe et al., 2017; Association for Pet Obesity Prevention, 2018; Öhlund et al., 2018). Clinically, obesity is considered a low-grade inflammatory disease, resulting from positive energy balance due to increased food intake, often a result of feeding regimen mismanagement by the owners, or reduced energy expenditure (German, 2006; Laflamme, 2006; German et al., 2010). However, many other factors contribute to obesity, including activity, breed, sex, neutering status, and age (Wall et al., 2019). Obese cats are also 3.9 times more likely to develop diabetes mellitus (Nelson and Reusch, 2014), a health condition characterized by insulin resistance, decreased glucose tolerance, and glucosuria.

In the wild, the cat is an opportunistic carnivorous hunter, feeding on small prey who themselves have different circadian rhythms, such as diurnal birds and nocturnal rodents, suggesting flexibility in the wild cats’ feeding patterns (Konecny, 1987). However, feeding rhythms may differ in domestic cats, which largely rely on humans for food provision. To investigate rhythms in food intake, domestic cats kept in L:D cycles (hours) of 10:14, 15:9, and 17:7, with *ad libitum* access to food and water, displayed rhythms in feeding behavior, albeit with a wide range of interindividual variability in nocturnal versus diurnal preference (Randall et al., 1985). About nocturnal feeding, there was a strong association with simulated nocturnal starlight as well as with human presence (Randall et al., 1985). As this study sought to determine percent nocturnality in cat food intake and activity, it was not clear whether the animals displayed multiple peaks in food intake throughout the day. Recently, Parker and colleagues investigated rhythms in food intake in a colony of 14 cats with *ad libitum* access to food, 7 of which showed a tendency toward bimodality, 4 being unimodal, and 3 being arrhythmic with peaks occurring between 0400-1000 h (dawn) and 1700-2100 h (dusk; Parker et al., 2019). While these studies appear to report contradicting results regarding cat feeding rhythms, they also reiterate that cats display interindividual variability and flexibility in their food intake, adapting to the environment around them. Further studies investigating the factors that determine diurnal versus nocturnal preference and bimodality are required.

In 2018, a consensus statement by the American Association of Feline Practitioners recommended frequent small meals throughout the day to support healthy body composition and weight, despite a lack of empirical evidence to support this regimen (Sadek et al., 2018). Indeed, a recent study reported that once-a-day feeding may promote satiation and development/maintenance of lean body mass in cats (Camara et al., 2020). In other reports, it has been shown that *ad libitum* access to food permits a cat to eat more than its energy requirements and may lead to increased body weight. Furthermore, *ad libitum* feeding relies on the presentation of dry food, which generally has a greater inclusion of carbohydrates compared to wet foods, since wet cat food cannot be provided for extended periods of exposure at room temperature (Michel et al., 2005). As cats are obligate carnivores, diets that are high in carbohydrates result in longer periods of postprandial hyperglycemia, which may lead to insulin resistance, a major risk factor for obesity and diabetes (Farrow et al., 2013). Therefore, strategies to reduce body weight that rely less on caloric restriction are warranted.

As discussed earlier in the section on dogs, TRE can provide significant cardiometabolic benefits, and it may also be an effective strategy for reducing excess body weight in cats. In a study by Deng and colleagues, cats were fed either twice daily (0800 and 2000 h) or four times daily (0800, 1200, 1600, and 2000 h) with commercial dry food. Cats fed twice daily showed more variability in glucose and insulin concentrations over 24 h and maintained higher insulin concentrations as compared to cats fed four times daily. In addition, cats fed 4 meals daily had consistently lower total ghrelin levels throughout the 24 h, while cats fed 2 meals daily had ghrelin concentrations above baseline during the light period. The opposite trend was observed for leptin concentrations. These findings suggest that cats fed more frequently with commercial dry food experience increased satiety compared to those fed less frequently (Deng et al., 2013).

However, a recent study by Camara and colleagues demonstrated contrasting results when cats were fed wet commercial food. Cats fed once a day (at 0800 h) had greater postprandial levels of appetite-regulating hormones such as gastric inhibitory polypeptide, glucagon-like peptide 1, and peptide YY compared to those fed four times a day (0800, 1130, 1500, and 1830 h). Cats fed once a day also showed lower postprandial respiratory quotients, which suggests greater fat oxidation occurring (Camara et al., 2020). Increasing feeding frequency has also been shown to decrease diurnal fluctuations in glucose, insulin, leptin, and ghrelin in cats (Deng et al., 2013). This may be due to more chronic exposure to dietary nutrients; however, the nutrient content of the diets is also a contributing factor to the physiological response. Over time, increased feeding frequency can lead to increased glucose tolerance, decreased insulin sensitivity, and eventual weight gain. Nevertheless, it suggests that feeding cats multiple meals throughout the day may disrupt entrainment by food or lead to circadian desynchrony, resulting in adverse metabolic profiles.

It is important to note that the mentioned studies did not examine feeding frequency during the dark period. Also, the cats were housed together although fed in a separate room; cohabitation should be considered as a variable in future research. Ultimately, these studies suggest that aligning feeding practices with cats’ circadian rhythms by providing food less frequently throughout the day could be beneficial in preventing and managing feline obesity and diabetes, especially when combined with appropriate lighting periods. However, there is a lack of available data to fully understand and differentiate these effects. Further research is needed to explore feeding times aligned with crepuscular, nocturnal, and diurnal patterns, as well as employing time-restricted food provision and alternating feeding frequencies.

## New frontiers

### Aquaculture

Aquaculture is the fastest growing food sector. For instance, in 2017 production was estimated at 180 million tons with more than 400 aquatic species farmed. It is predicted that by 2050, production will be bolstered by an additional 100 million tons of seafood, mostly from cultured species (Stentiford et al., 2017, 2022). Necessary intensification of the industry poses significant health and welfare challenges and wider environmental issues. In fish, particularly salmonids such as Atlantic salmon, *Salmo salar*, it is now common practice to use highly extended day lengths or even L:L to increase growth (Boeuf and Le Bail, 1999) and manipulate maturation (Strand et al., 2018) and reproduction (Wang et al., 2010). While in some species, including Atlantic salmon, these extreme light regimes appear to have no observable behavioral or physiological effects (Fang et al., 2019; Hines et al., 2019; Hamilton et al., 2022), in others such as Coho salmon (*Oncorhynchus kisutch*) and rainbow trout (*Oncorhynchus mykiss*), they elicit markers of stress and/or alter immune profiles (Melingen et al., 2002; Leonardi and Klempau, 2003). However, the extent to which manipulation of circadian biology in cultured aquatic species influences disease susceptibility—arguably the greatest challenge to aquaculture (Stentiford et al., 2017, 2022)—is largely unknown.

In common with other vertebrates, fish have cycling immunity (Lazado et al., 2016; Onoue et al., 2019; Zhang et al., 2020). However, they have decentralized clocks (Froland Steindal and Whitmore, 2019) and perturbation of these peripheral clocks and consequent disruption of rhythmic immunity could expose vulnerabilities in the host fish to pathogenic attack or parasitism. Indeed, it has recently been shown that L:L disrupts immune gene expression in the skin of rainbow trout and has negative impacts on resistance to lice infestation (Ellison et al., 2021). Intriguingly, in the same study, skin microbiome profiles which also showed daily modulation in abundance and diversity are concomitantly perturbed by L:L. Given the association of microbial communities and immunological status in other mammalian models (Thaiss et al., 2016), L:L might have detrimental consequences not yet revealed with more routine physiological or behavioral analyses.

Fish photobiology and clock gene biology are very complex, particularly teleost fish that have undergone full genome duplications. For example, zebrafish have been shown to have 42 distinct photopigment genes including 5 melanopsin genes (Davies et al., 2015). Nevertheless, recognition and deeper appreciation of the nuances of circadian biology on fish health could yield considerable benefits from a productivity and welfare perspective, contributing to a more sustainable industry. For example, chronotherapeutic approaches to pathogen control and disease mitigation could benefit the producer in terms of efficacious dosing and have positive ecological impacts as have been shown in other vertebrate and even plant systems (Belbin et al., 2019). Moreover, TRE strategies are gaining interest in terms of salmonid growth and feed efficiencies (the single greatest cost to producers; Asche and Oglend, 2016). While TRE is known to affect immunity and other health parameters in mammalian models (Geiger et al., 2017; Di Francesco et al., 2018; Zheng et al., 2020), this is largely unexplored in cultured aquatic species. Vitally, chronotherapeutic and chrononutrition practices must consider inter-specific differences; matching conditions to chronotypes to enhance welfare, growth, and viability.

### Preserving Circadian Rhythms in Veterinary Hospitals and ICU: A Rationale for Optimal Patient Care

#### Lighting and Circadian Disruption in Veterinary Hospitals

In recent decades, the management of human patients in ICUs has intensified, inadvertently leading to increased environmental stimuli like light. In humans, it has been shown that these disruptions can disturb patients’ circadian rhythms and sleep, potentially compromising the healing process (Drouot et al., 2008; Buxton et al., 2012; Ruben et al., 2019a, 2019b). This disruption similarly affects animals. Experimental studies using rodent models of heart disease have shown how a disturbed L:D environment, similar to ICU conditions, impairs recovery and healing. For example, disrupting circadian rhythms immediately following a myocardial infarction (heart attack) in mice severely impairs inflammatory responses, hindering healing (Alibhai et al., 2014). These findings highlight the detrimental effects of circadian disruption on patient outcomes (Alibhai et al., 2015; Reitz and Martino, 2015; Khaper et al., 2018). However, there has been limited focus on how this affects companion animal patients in veterinary hospitals.

Veterinary critical care units function similarly to human hospitals, providing intensive, around-the-clock care in well-lit environments. Just as seriously ill humans, critically ill animals in veterinary hospitals often need extended stays for treatments—ranging from chemotherapy complications to acute kidney injury, spanning several days to weeks (Britton et al., 2014) and 3-48 days for acute kidney injury (Thoen and Kerl, 2011). While no specific studies have investigated light disturbance effects on cats and dogs in veterinary hospitals, evidence suggests that these species are physiologically impacted by circadian disruptions. Studies indicate that varying light intensities affect dogs’ sleep behavior, with low light intensity improving sleep and strong illumination causing varied effects (Fukuzawa and Nakazato, 2015). L:L exposure has disrupted rhythmicity in measures like intraocular pressure in dogs (Piccione et al., 2010), and cerebral spinal fluid concentrations of vasopressin and melatonin in cats (Reppert et al., 1982). Consequently, animal patients in these critical care units might experience disturbances in their circadian rhythms and sleep due to L:L exposure, mirroring the effects observed in human patients. Therefore, a promising area for future circadian medicine applications is in normalizing the light and dark period for animals in critical care environments.

#### Non-photic Disruptions in Veterinary Hospitals: Sound and Smell

In veterinary hospitals, non-photic disruptions like noise and smell might significantly affect animal patients, possibly more than humans. Cats and dogs have enhanced sensory sensitivity, especially to auditory and olfactory stimuli, and often experience fear-related issues during clinic visits and veterinary procedures. Their wider hearing range (Heffner, 1983; Heffner and Heffner, 1985) makes them more sensitive to high-frequency noises commonly found in veterinary settings, such as electronic devices (e.g., monitoring equipment, computer screens) and mechanical noises (e.g., kennel doors opening and closing, equipment being moved around). This suggests that noise in veterinary hospital environments might impede animals’ healing processes.

Notably, cats and dogs exhibit evident stress responses to loud noises in general (Gruner, 1989; Haverbeke et al., 2008; Eagan, 2020) and during veterinary visits (Stellato et al., 2019; Furgala et al., 2022). They are prone to noise phobia (Blackwell et al., 2013; Storengen and Lingaas, 2015; Gates et al., 2019) and exhibit fear-related behaviors. Consequently, sound becomes an important disruptor to consider in veterinary critical care units. A study conducted in academic veterinary ICU settings found that noise levels exceeded the recommendations by the World Health Organization of a maximum of 35 dB at night and in human ICUs (Fullagar et al., 2015), with average levels between 1800 and 2100 h above 77 dB, levels reaching the equivalent of a vacuum cleaner or an average radio (Dornbusch et al., 2020).

Moreover, cats and dogs possess highly sensitive olfactory systems (Vitale Shreve and Udell, 2017; Kokocińska-Kusiak et al., 2021). Exposure to various odors, such as cleaning supplies or pheromones, can trigger fear and general disturbance. Despite this, there is limited research on how these smells impact animals in veterinary settings, highlighting the need for further investigation. Overall, non-light disruptions, especially noise and smell, might significantly impact companion animals more than human patients, necessitating careful consideration in veterinary care. This understanding of the animals’ stress and fear responses during veterinary care can guide approaches that aim to minimize these stressors, potentially leading to improved patient comfort, faster recovery times, and better overall health outcomes.

#### Non-photic Disruptions—Fear and Stress

Animal patients also experience high levels of fear and stress during veterinary visits. Studies on rodents suggest that fear experiences can impact circadian rhythms (e.g., Amir and Stewart, 1998; Pellman et al., 2015), indicating the potential for stressors to exacerbate circadian disruptions in veterinary critical care units. Animal patients are exposed to a range of stressors in the clinic including unfamiliar environments, noises, odors, people, and other animals, as well as handling and restraint and uncomfortable and painful procedures. While these experiences are also common for humans during hospital visits, animals lack the same understanding of the necessity for their visit and generally have limited predictability and control of their experiences during care. As a result, most cats and dogs show increased signs of fear and stress during standard clinic visits, during routine handling, and procedures (Stanford, 1981; Döring et al., 2009; Glardon et al., 2010; Moody et al., 2020). This heightened stress can lead to increased disturbance during routine monitoring and procedures, as well as ongoing hypervigilance even during undisturbed periods.

#### Effective Strategies for Enhancing Animal Welfare and Healing

In veterinary intensive care, patients need continual attention regardless of day or night. To minimize circadian disruptions without compromising critical care, adopting strategies like cycle lighting and light-blocking at night can be beneficial, drawing from successful approaches used in human critical care units. This includes implementing a “chrono-bundle” of interventions (Scotto et al., 2009; Xie et al., 2009; Hu et al., 2010; Patel et al., 2014; McKenna et al., 2018), potentially employing specific light spectra, such as blue-poor light during sleep time to limit light-induced melatonin suppression (Souman et al., 2018). These strategies, proven to reduce sleep disruptions and delirium in human hospitals (Engwall et al., 2015, 2017; Ruben et al., 2019a), may offer similar benefits to animal patients. Moreover, controlling stimuli like noise and activity during rest periods, possibly through separating emergency triage areas from recovery spaces, could decrease stress and promote better circadian health in recovering animals. Further research into these practices could greatly enhance veterinary care.

## One health and conclusions

Embracing the One Health concept acknowledges the interconnection between human, non-human animal, and environmental health (Zinsstag et al., 2011; Ancheta et al., 2021a, 2021b; Kelley, 2022). By recognizing the shared health challenges between species, disciplines like human and veterinary medicine can collaborate to optimize health outcomes for all.

Circadian rhythms, crucial for mammalian well-being, are intricately tied to environmental cues like light and food. Disruptions to these rhythms, often due to altered light environments, have been associated with various health issues, including metabolic instability and diseases like obesity, diabetes, cardiovascular conditions, cancer, and other pathologies. Understanding and addressing these disruptions play a vital role in promoting comprehensive well-being across species within the One Health framework.

Recent scientific advances in circadian medicine, involving innovative approaches like TRE, chronotherapy, and circadian lighting, have shown promise for human health. However, there is a notable gap in exploring these advancements for animals. By adopting a One Health perspective, collaborative efforts can drive the development of novel therapies benefiting not only humans, but also non-human animal health. This holistic approach emphasizes collective efforts to tackle shared health challenges, transcending conventional boundaries and leading to a healthier future for all.
